# Use of saliva as an alternative diagnostic method for diagnosis of COVID-19

**DOI:** 10.1016/j.ijregi.2023.03.011

**Published:** 2023-05-04

**Authors:** Meutia Wardhanie Ganie, Irbah Rea Alvieda Nainggolan, Ramadhan Bestari, Al Hamidy Hazidar, Mirzan Hasibuan, Jelita Siregar, Muhammad Ichwan, R. Lia Kusumawati, Inke Nadia Diniyanti Lubis

**Affiliations:** a Faculty of Medicine, Universitas Sumatera Utara, Medan, Indonesia; b Faculty of Medicine, Universitas Islam Sumatera Utara, Medan, Indonesia; c Faculty of Computer Science and information Technology, Universitas Muhammadiyah Sumatera Utara, Medan, Indonesia; d Institute IR 4.0, Universiti Kebangsaan Malaysia, Bangi, Malaysia; e Department of Microbiology, Faculty of Medicine, Universitas Sumatera Utara, Medan, Indonesia; f Department of Clinical Pathology, Faculty of Medicine, Universitas Sumatera Utara, Medan, Indonesia; g Department of Pharmacology, Faculty of Medicine, Universitas Sumatera Utara, Medan, Indonesia; h Department of Paediatrics, Faculty of Medicine, Universitas Sumatera Utara, Medan, Indonesia; i Menzies School of Health Research, Darwin, Australia

**Keywords:** COVID-19, Coronavirus, Saliva, SARS-CoV-2, Diagnosis, Indonesia

## Abstract

**Background:**

Mass population testing has been recommended to contain the spread of severe acute respiratory syndrome coronavirus-2 (SARS-CoV-2) infection. However, the use of nasopharyngeal swab specimens has caused many logistic challenges. This study investigated the sensitivity and specificity of saliva as a non-invasively-obtained specimen for molecular detection of SARS-CoV-2 RNA.

**Methods:**

In total, 153 patients with confirmed coronavirus disease 2019 (COVID-19) who had been admitted to the regional referral hospital or who self-isolated at home were included in this study. Nasopharyngeal swab specimens and saliva samples were collected on the same day, and were tested for SARS-CoV-2 infection using reverse transcriptase polymerase chain reaction.

**Results:**

The sensitivity and specificity of saliva samples were 81.5% and 76.4%, respectively, in cases that had been confirmed as COVID-19 using nasopharyngeal swab samples. Positive predictive values and negative predictive values were 92.3% and 54.1%, respectively. The highest detection rates were found among samples collected 4–7 days since symptom onset.

**Conclusion:**

Saliva samples showed comparable performance to nasopharyngeal swab specimens for the diagnosis of COVID-19 in adults. The performance of saliva as a diagnostic specimen for COVID-19 testing is particularly significant during the first week of symptoms.

## Highlights

• Saliva can be used an alternative specimen for the diagnosis of coronavirus disease 2019.
• Standardized methods for the collection of saliva samples are needed.
• The performance of saliva is higher during the first week after symptom onset.
• Sensitivity and specificity of saliva were 81.5% and 76.4%, respectively.
• Individuals with upper-respiratory-tract-infection-related symptoms are more likely to test positive using saliva samples.

## Introduction

Coronavirus disease 2019 (COVID-19) is an infectious disease caused by severe acute respiratory syndrome coronavirus-2 (SARS-CoV-2), which emerged in China and subsequently spread worldwide [1]. Since being declared a global pandemic, COVID-19 has overwhelmed many healthcare systems. Although substantial progress in clinical research has led to a better understanding of SARS-CoV-2 and the management of COVID-19, limiting continuation of the spread of this virus and its variants has become an issue of increasing concern [2]. Most countries tried to contain the spread of SARS-CoV-2 by implementing numerous control measures, including early diagnosis and mass population testing. Early diagnosis is critical to prompt actions on patient management, infection control and public health control measures, while mass testing, rigorous contact tracing, quarantine and isolation have been recommended to halt the pandemic [3]. However, the preparedness of countries to implement these strategies varies. There are limitations facing the COVID-19 pandemic, including limited costs and funding for COVID-19 diagnosis, restricted logistics provision, non-availability of standard polymerase chain reaction laboratories, and a shortage of experts. This affects each country's overall readiness to face the COVID-19 pandemic.

Nasopharyngeal and oropharyngeal swab specimens are usually considered to have the highest diagnostic sensitivity, and have been recommended by the World Health Organization. Collection of these specimens is invasive, causes discomfort to the patient, and requires significant manpower and personal protective equipment [4]. To solve these logistical challenges and the limited manpower associated with specimen collection, there is a need to find a specimen that can be collected easily and which can be used as a substitute for nasopharyngeal swab specimens, and saliva is currently attracting considerable interest as a specimen for SARS-CoV-2 detection [5]. The use of saliva as a diagnostic sample has several advantages: saliva can be provided easily by the patient, and collection is more comfortable compared with nasopharyngeal swab and sputum procedures [6]. A previous study of SARS-CoV-2 diagnosis using posterior oropharyngeal saliva, in which patients were asked to spit into a sterile container, found that the sensitivity of saliva for diagnosis was high [7]. As such, the present study aimed to compare nasopharyngeal swab and saliva specimens for the molecular detection of SARS-CoV-2 RNA among patients with COVID-19 in Indonesia.

## Methods

A cross-sectional study was conducted between June and December 2020 to compare the use of nasopharyngeal swab specimens and saliva samples for the detection of SARS-CoV-2 using reverse transcriptase polymerase chain reaction (RT-PCR). The inclusion criteria were: patients aged ≥18 years who had been confirmed to have COVID-19 by RT-PCR using nasopharyngeal swab specimens; patients hospitalized at one of the following hospitals –Martha Friska Hospital, Pirngadi General Hospital, Murni Teguh Hospital, Santa Elisabeth Hospital or Royal Prima Hospital in Medan, Indonesia – or who self-isolated at home; and patients for whom both saliva and nasopharyngeal swab specimens were collected within 4 h of each other.

In total, 153 pairs of nasopharyngeal swab and saliva specimens were collected from 153 patients. Saliva was self-collected, with patients spitting 3–5 mL of saliva directly into a sterile bottle in the early morning before rinsing their mouth or eating breakfast. The nasopharyngeal swab specimen for each patient was collected by trained personnel using a flocked swab within 4 h of collection of the saliva specimen, and this was immersed in 2 mL of viral transport medium, as described previously [8].

All RT-PCR examinations were undertaken in the Microbiology Laboratory, Faculty of Medicine, Universitas Sumatera Utara, Medan, Indonesia. RNAs from both specimens were extracted manually with a spin column using an RNA Purification Kit (Guangzhou Da Te Bioengineering Technology Co., Ltd, Guangzhou, China), in accordance with the manufacturer's instructions. The master mix was prepared by mixing 17 μL of NC (ORF1ab/N) PCR liquid A (reaction mix) and 3 μL of NC (ORF1ab/N) PCR reaction liquid B (enzyme) from the Da An Gene Co.Cat.#DA-930 reagent. Five microlitres of the extracted sample was added to make a final volume of 25 μL. The RNA plate was loaded immediately on to the Roche LC-96 and LC-480, and the probe detection modes were set as: open reading frame 1ab gene (ORF1ab): VIC; nucleocapsid gene (N gene): FAM; and RNA-polymerase gene (internal control): Cy5. The PCR cycle was carried out as follows: one 15-min cycle at 50°C, one 15-min cycle at 95°C, and 45 cycles of 94°C for 15 s and 55°C for 45 s. The cycle threshold (Ct) value of the RT-PCR was used as an indicator of the copy number of SARS-CoV-2 RNA specimens, with lower Ct values corresponding to higher viral copy numbers. A Ct value <40 was considered positive for SARS-CoV-2 RNA.

RT-PCR results were recorded on an Excel spreadsheet (Microsoft Corp., Redmond, WA, USA). Data were analysed for descriptive were presented as *n* (%) for categorical variables and median. Univariate, bivariate and multivariate logistic regression and ANOVA analyses were used to assess the relationship between gender, age, clinical onset and severity with the PCR results from the saliva specimen and viral load. *P*<0.05 was considered to indicate significance. Sensitivity, specificity, positive predictive value and negative predictive value were calculated to assess diagnostic performance. All statistical analyses were performed using SPSS Version 23.0 (IBM Corp., Armonk, NY, USA).

All study subjects received an information sheet and were asked to provide signed informed consent. This study was approved by the Research Ethics Committee of the Faculty of Medicine, Universitas Sumatera Utara (Ref. No. 716/KEP/USU).

## Results

In total, 153 patients were included in this study. Of these, 53.6% were female, the median age was 43 years [interquartile range (IQR) 33–55], 88.2% were hospitalized, and 58.2% were tested during the first week since symptom onset (Table 1). Sixty-five percent of patients had mild symptoms, 73.9% had respiratory symptoms, and 9.8% were asymptomatic throughout their infection. The median Ct value for positive samples using nasopharyngeal swab specimens was 32.3 (IQR 26.3–36.3). No significant differences were found between the proportion of Ct values ≤30 and >30 at different time points since symptom onset (*P*>0.05).

**Table 1:** Characteristics of patients under investigation diagnosed with coronavirus disease 2019 by reverse transcriptase polymerase chain reaction using nasopharyngeal swab and saliva specimens.

Characteristics	*n* (%)
Sex	
Male	71 (46.4)
Female	82 (53.6)
Age group (years) 19–35 36–55 >55	46 (30.1) 70 (45.8) 37 (24.2)
Type of isolation Hospitalized Self-isolation	107 (88.2) 46 (11.8)
Clinical symptoms Cough Fever Dyspnoea Headache Sore throat Cold Weakness Nausea/vomit Anosmia Diarrhoea Asymptomatic	94 (28.4) 66 (19.9) 38 (11.5) 36 (10.9) 23 (6.9) 21 (6.3) 21 (6.3) 13 (3.9) 11 (3.3) 8 (2.4) 18 (11.8)
Type of symptoms Acute respiratory tract infection	113 (73.9)
Non-acute respiratory tract infection	25 (16.3)
Asymptomatic	15 (9.8)
Disease severity Asymptomatic Mild Moderate	15 (9.8) 100 (65.4) 38 (24.8)
Days of testing since clinical onset*	
Median number of days (IQR) ≤3 4–7	6 (3–10) 41 (26.8) 48 (31.4)
7–14 >14	43 (28.1) 21 (13.7)
Ct values of nasopharyngeal swab specimens Median Ct (IQR) ≤30 >30 Ct values of saliva specimens Median Ct (IQR) ≤30 >30	32.6 (26.6–36.7) 45 (37.5) 75 (62.5) 29.7 (25.2–35.0) 53 (50.4) 52 (49.5)

IQR, interquartile range; CT, cycle threshold.

*Days since the first positive result was used for asymptomatic patients

Of 153 patients, 83% (127) had either nasopharyngeal swab or saliva specimens that tested positive using RT-PCR. Of these, 63.3% (97/153) of patients tested positive for both nasopharyngeal swab and saliva specimens, 14.4% (22/153) of patients tested positive for nasopharyngeal swab specimens alone, and 5.2% (8/153) of patients tested positive for saliva specimens alone (Table 2). The RT-PCR results using nasopharyngeal swab specimens showed higher positivity rates compared with saliva samples (77.8% vs 67.9%; *P*=0.001). Significantly higher positivity rates were observed from nasopharyngeal swab specimens tested ≤3 days, 8–14 days and >14 days since symptom onset (*P*=0.01), but not on those tested 4–7 days since symptom onset when the highest detection rates were seen for saliva samples (81.3%; *P*=0.198). The detection rates of saliva samples reduced to 58.2% and 52.4% for samples tested 8–14 days and >14 days since symptom onset, respectively (*P*=0.04).

**Table 2:** Comparison between nasopharyngeal swab and saliva specimens for the detection of severe acute respiratory syndrome coronavirus-2 infection according to days since symptom onset.

Days since symptom onset	RT-PCR results				*P-*value^a^	Kappa
	Saliva	NP swab				
		Positive		Negative		
All samples at any time	Positive	96 (62.7%)	8 (5.2%)		0.001	0.493
	Negative	23 (15.0%)	26 (16.9%)			
≤3 days	Positive	27 (65.8%)	2 (4.9%)		0.007	0.396
	Negative	5 (12.2%)	7 (17.1%)			
Days 4–7	Positive	36 (75.0%)	3 (6.3%)		0.198	0.175
	Negative	7 (14.5%)	2 (4.2%)			
Days 8–14	Positive	23 (53.5%)	2 (4.7%)		0.001	0.499
	Negative	8 (18.6%)	10 (23.3%)			
>14 days	Positive	10 (47.6%)	1 (4.8%)		0.001	0.809
	Negative	1 (4.8%)	9 (42.9%)			

RT-PCR, reverse transcriptase polymerase chain reaction.

a Chi-squared analysis

Individuals with non-acute respiratory tract infections [odds ratio (OR) 0.27, 95% confidence interval (CI) 0.10–0.973; *P*=0.01] and Ct values >30 (OR 0.11, 95% CI 0.01–0.250; *P*=0.01) were less likely have positive RT-PCR results with saliva specimens, while individuals aged between 36 and 55 years and >55 years were more likely to have positive RT-PCR results with saliva specimens (OR 3.12, 95% CI 1.31–7.46, *P*=0.01; OR 3.11, 95% CI 1.10–9.12, *P*=0.02). After adjusting for significant risk factors, none of the risk factors were significantly associated with a positive RT-PCR result with saliva specimens (Table 3).

**Table 3:** Analysis between demographic characteristics and saliva reverse transcriptase polymerase chain reaction (RT-PCR).

Demographic	Saliva RT-PCR results		OR*	95% CI	*P-*value	aOR^†^	95% CI	*P*-value
Positive	Negative							
Sex								
Male	50	21	Ref					
Female	54	28	0.81	0.38–1.69	0.55			
Age group (years) 19–35 36–55 >55	23 53 28	23 17 9	Ref 3.12 3.11	1.31–7.46 1.10–9.12	0.01 0.02	Ref -1.81 -1.20	-0.33–0.15 -0.32–0.08	0.07 0.23
Type of symptoms Acute respiratory tract infection	84	29	Ref			Ref		
Non-acute respiratory tract infection Asymptomatic	11 9	14 6	0.27 0.52	0.10–0.73 0.15–1.94	0.01 0.24	1.67 0.22	-0.03–0.39 -0.23–0.28	0.09 0.82
Disease severity Asymptomatic Mild Moderate	9 68 27	6 32 11	0.71 Ref 1.16	0.20–2.64 0.48–2.91	0.53 0.72			
Days of testing since symptom onset*								
≤3 4–7	29 35	12 9	Ref 1.61	0.53–4.96	0.34			
7–14 >14	25 11	18 10	0.57 0.45	0.20–1.55 0.13–1.56	0.22 0.15			
Ct value of nasopharyngeal swab ≤30 >30	43 53	2 22	Ref 0.11	0.01–0.50	0.01	Ref 12.27	0.95–1.31	0.001

Ct, cycle threshold.

^a^Logistic regression analysis.

^b^Multivariate analysis of variance, adjusted for age, type of symptoms and Ct values of nasopharyngeal swab specimen.

Nasopharyngeal swabs were used as the reference standard to determine the diagnostic test performance of saliva for SARS-CoV-2 RT-PCR. The sensitivity and specificity of saliva samples were 80.7% and 76.5%, respectively, and positive predictive values and negative predictive values were 92.3% and 53.1%, respectively (Table 4).

**Table 4:** Diagnostic values of saliva specimens against nasopharyngeal swab specimens using reverse transcriptase polymerase chain reaction for severe acute respiratory syndrome

Saliva specimen	Diagnostic test performance					
	Sensitivity	Specificity	PPV	NPV	LR+	LR-
	80.7%	76.5%	92.3%	53.1%	3.43	0.25

PPV, positive predictive value; NPV, negative predictive value; LR+, positive likelihood ratio; LR-, negative likelihood ratio.

## Discussion

This study showed the value of using saliva as a non-invasive method for detection of SARS-CoV-2 infection. Saliva is a biofluid that can be obtained with minimal discomfort and adequate safety in the context of the COVID-19 pandemic. RT-PCR test results for saliva specimens showed differing sensitivity and performance compared with nasopharyngeal swab specimens. In Indonesia, the Ministry of Health has approved several specimens and diagnostic methods for use for SARS-CoV-2 diagnostic purposes, including blood, naso-oropharyngeal swabs, saliva and volatile organic compounds [9]. Saliva samples have been reported to be superior to nasopharyngeal swab specimens, especially after symptom onset, when collected using the passive drool method to obtain a homogenous specimen and to avoid the influence of inhibitory substances [6,10]. In other studies, saliva samples were collected after coughing, and individuals were asked to spit several times into a sterile pot; this resulted in moderate to high performance of saliva samples in comparison with nasopharyngeal swab specimens [11,12]. In the present study, saliva samples were found to have lower sensitivity than nasopharyngeal swab specimens, and saliva samples were only comparable to nasopharyngeal swab specimens when tested within 4–7 days of symptom onset.

In the present study, saliva samples were self-generated by the patients, without the need for coughing. Using this sampling method, saliva samples were found to be moderately comparable with nasopharyngeal swab specimens. However, the range of sensitivities found using saliva samples suggests the need for more standardized and validated methods, and at least three conditions should be optimized [13]: different saliva collection methods should be compared systematically to select the optimal method; the optimal solution for collecting, transporting and storing saliva samples should be identified; and the RNA assay method (either quantitative RT-PCR, loop-mediated isothermal amplification or another protocol) should be optimized for saliva using an appropriate internal control. With this validation, the results of RT-PCR using saliva samples collected with the optimal method can be expected to produce stable results that are comparable with the results of nasopharyngeal swab specimens.

This study showed that individuals with non-acute-respiratory-tract-infection-related symptoms were less likely to have positive RT-PCR results using saliva samples, and this was not associated with time since symptom onset or Ct value. Over 70% of individuals with COVID-19 present with lower and upper respiratory symptoms [14], similar to the findings of the present study. ACE-2 epithelial cells of the salivary glands and respiratory systems are the initial targets of SARS-CoV-2. Once the virus gains entry into the host's cells, viral replication results in direct virus-mediated tissue damage, and the infected host cells subsequently trigger local and systemic immune responses [2,15]. The source of SARS-CoV-2 in saliva is unknown, but it could come from multiple locations, including secretion into the saliva from the glands, and from other sources such as infected oral mucosal endothelial cells, and the lower and upper respiratory tract [4,16,17]. This explains the higher probability of positive RT-PCR results for saliva in individuals with respiratory symptoms.

This study had a few limitations. First, samples were collected from patients with a median time since symptom onset of 6 days. Previous studies have shown that the Ct value is highest during the first week since symptom onset, and this contributed to higher sensitivity of RT-PCR using saliva samples [18], [19], [20]. However, the majority of saliva samples in the present study had Ct values >30, with a similar proportion of samples with low and high Ct values at different time points since symptom onset. Individuals were enrolled consecutively during the study period, and therefore the findings may show that the SARS-CoV-2 viral load among mild-to-moderate cases of COVID-19 was low. This led to the second limitation of this study – individuals with severe and critical symptoms were not enrolled. Therefore, this study may not reflect the sensitivity of saliva for individuals with more severe symptoms.

In conclusion, saliva is a non-invasive diagnostic specimen that is moderately comparable with nasopharyngeal swab specimens for the detection of SARS-CoV-2. The use of saliva samples is preferred in situations where collection of nasopharyngeal and oropharyngeal swabs is difficult or should be avoided, and saliva can certainly be used as a tool for self-collection. However, there are variations in sensitivity when saliva is used for diagnosis, especially when different types of sampling methods are used. As such, future studies should aim to standardize the sampling method for saliva in order to produce stable and RT-PCR results.

## Funding

This study was supported by the Indonesian Ministry of Education and Culture Universitas Sumatera Utara (World Class Research 2020 Scheme 1879/UN5.1.R/SK/PPM/2020) and received a financial support from Universitas Sumatera Utara (ICTROMI Funding).

## Conflict of interest statement

None declared.

## Transparency declaration

This article is part of a supplement entitled ‘Proceedings from the 3rd International Conference on Tropical Medicine & Infectious Diseases’ published with support from the Universitas Sumatera Utara, Medan, Indonesia.
